# Extracorporeal membrane oxygenation support for four patients with acquired immunodeficiency syndrome complicated by pneumocystis pneumonia: a case series and literature review

**DOI:** 10.3389/fmed.2026.1804787

**Published:** 2026-07-23

**Authors:** Yao Sun, Jingjing Hao, Yufeng Liu, Xiaoxi Zhong, Xueqing Wang, Lin Pu, Jingyuan Liu

**Affiliations:** Beijing Ditan Hospital, Capital Medical University, Beijing, China

**Keywords:** acute respiratory failure, AIDS, extracorporeal membrane oxygenation, mechanical ventilation, PCP, pneumomediastinum, spontaneous pneumothorax, ventilator-induced lung injury

## Abstract

**Background:**

Acquired immunodeficiency syndrome (AIDS) complicated by Pneumocystis pneumonia (PCP) is characterized by an insidious onset, rapid progression, and a critical clinical course. Severe complications, such as respiratory failure and pneumothorax, may occur in the absence of timely and effective intervention, resulting in increased mortality. Extracorporeal membrane oxygenation (ECMO) can serve as a salvage therapy by temporarily and effectively replacing cardiopulmonary function.

**Objective:**

We aimed to summarize the clinical experience of veno-venous extracorporeal membrane oxygenation (V-V ECMO) support in four patients with AIDS complicated by PCP.

**Methods:**

Clinical data from four patients with AIDS who were complicated by PCP and received ECMO support at Beijing Ditan Hospital, Capital Medical University, were retrospectively analyzed. The timing of ECMO initiation, mode selection, mechanical ventilation (MV) management, sedation and analgesia strategies, prognosis, and clinical experience were evaluated, and treatment experience was summarized.

**Results:**

All four patients were definitively diagnosed with AIDS complicated by PCP and respiratory failure. All patients received ventilatory support and developed barotrauma. Pneumothorax occurred in cases 1, 2, and 4, whereas pneumomediastinum was detected in case 3. All patients received V-V ECMO support, which improved clinical conditions in all cases. Finally, cases 1 and 2 died, whereas cases 3 and 4 recovered and were discharged. Among them, awake ECMO was successfully implemented in case 3.

**Conclusion:**

For patients with AIDS complicated by PCP, particularly for those with concomitant barotrauma, early initiation of ECMO support may rapidly improve systemic oxygenation and lower the intensity of MV. This approach may decrease ventilator-induced lung injury (VILI), facilitate lung rest and lung protection, and provide a therapeutic window for treating the underlying disease. Clinicians should comprehensively evaluate disease reversibility, immune recovery potential, and the severity of lung injury. Withholding ECMO therapy solely due to HIV infection status should be avoided.

## 1 Introduction

PCP is an interstitial pneumonia caused by infection with *Pneumocystis jirovecii*. It primarily affects immunocompromised individuals. The radiological manifestations of this disease include pulmonary cysts, pneumatoceles, and spontaneous pneumothorax (1). In patients with AIDS, PCP represents a severe opportunistic infection. A single-center retrospective study conducted at Beijing YouAn Hospital included 972 eligible patients with AIDS complicated by PCP and reported an overall in-hospital mortality rate of 17.8% (2). PCP is characterized by an insidious onset, rapid progression, and a critical clinical course. Severe complications, such as respiratory failure and pneumothorax, may occur in the absence of timely and effective treatment, resulting in increased mortality (3).

Compared with the general population, patients with AIDS are at a higher risk of spontaneous pneumothorax, which is estimated to affect approximately 2%–5% of all cases (4). In patients with AIDS, the successful treatment of pneumothorax remains challenging. HIV-infected patients who develop pneumothorax generally exhibit a poor prognosis, with a complication-related mortality rate ranging from 30% to 60% (4). Afessa et al. reported an in-hospital mortality rate of 30.8% among HIV-infected patients with pneumothorax, compared with 5.8% among HIV-infected patients without pneumothorax (*p* = 0.0060) (5). In a small case series, Gomez et al. estimated that the mortality rate of HIV-infected patients with pneumothorax remains as high as 66%, with a mean survival length of 55.6 days (6). This poor outcome is mainly attributable to the fact that spontaneous pneumothorax in these patients is often accompanied by severe subpleural necrotic lesions. The lung parenchyma is inflamed and fragile in such patients, preventing complete lung re-expansion. Therefore, persistent pneumothorax cannot be resolved through closed thoracic drainage. In addition, MV further decelerates pneumothorax healing, thereby prolonging the required duration of drainage for recovery.

Currently, worldwide experience in the management of patients with AIDS complicated by respiratory failure and persistent pneumothorax remains limited, and clinical management still faces considerable challenges. To prevent organ damage caused by hypoxia, ECMO can be adopted as a salvage therapy to temporarily and effectively replace cardiopulmonary function when MV fails to maintain adequate oxygenation. By providing gas exchange while allowing lung-protective ventilation, ECMO improves oxygenation and contributes to cardiopulmonary rest, making it a feasible adjunctive therapeutic modality. In this study, we retrospectively analyzed four patients with AIDS complicated by PCP who received ECMO at Beijing Ditan Hospital, Capital Medical University. The timing of ECMO initiation, mode selection, MV management, sedation and analgesia strategies, prevention of complications, prognosis, and clinical experience were evaluated, and treatment experience was summarized. This study may optimize the application of ECMO technology in patients with AIDS and provide additional options for respiratory support in patients with PCP complicated by pneumothorax or at a high risk of pneumothorax.

## 2 Case presentation

### 2.1 Case 1

The patient was a 25-year-old man with a height of 180 cm and a body weight of 55 kg who was previously healthy. The patient had a history of homosexual intercourse and was admitted to the Department of Infectious Diseases of Beijing Ditan Hospital, Capital Medical University, on October 18, 2017, because of “dyspnea for 1 month and newly detected positive HIV antibody for 1 day.” The patient had never received antiretroviral therapy (ART). His CD4^+^ T-cell count was 5 cells/μL, and his HIV RNA level was 4.8^E+5^ copies/mL. Chest computed tomography (CT) showed diffuse ground-glass opacities in both lungs. Pathological examination of BALF demonstrated morphological features consistent with PCP under high-power microscopy, and Gomori methenamine silver staining was positive. The patient was diagnosed with AIDS based on positive HIV antibody screening and qualitative HIV nucleic acid testing in the presence of an opportunistic infection. PCP was diagnosed based on the characteristic radiological findings and BALF pathological examination. His oxygen saturation (SpO_2_) was 90%, suggesting respiratory failure. After admission, the patient received SMZ-TMP [each tablet containing sulfamethoxazole (SMZ) 400 mg and trimethoprim (TMP) 80 mg] combined with clindamycin for the treatment of PCP. In addition, glucocorticoids were administered to reduce inflammatory exudation. Moreover, oxygen therapy was conducted via face mask at a flow rate of 10 L/min. During hospitalization, ART (bictegravir sodium, emtricitabine, and tenofovir alafenamide fumarate tablets) was initiated.

On hospital day 16, chest CT revealed a small right-sided pneumothorax. On hospital day 27, the patient developed worsening dyspnea after straining during defecation. His SpO_2_ decreased to 60%, and his respiratory rate increased to 40 breaths/min. He was transferred to the intensive care unit (ICU), where emergency endotracheal intubation and MV were initiated. Chest radiography revealed bilateral pneumothorax and pneumomediastinum. Bilateral closed thoracic drainage and tracheostomy were subsequently conducted. Sedation, analgesia, and neuromuscular blockade were also administered. The ventilator was set to the pressure assist/control (P-A/C) mode with an FiO_2_ of 45%, a positive end-expiratory pressure (PEEP) of 6 cmH_2_O (1 cmH_2_O = 0.098 kPa), a pressure control (PC) threshold of 22 cmH_2_O, a respiratory rate of 25 breaths/min, and a monitored tidal volume (Vt) of 340 mL. The patient was also complicated by septic shock and received empirical antibiotics, fluid resuscitation, and norepinephrine infusion (0.5–1.0 μg/kg/min) for circulatory support. Due to the high respiratory support demand and poor healing of bilateral pneumothorax, V-V ECMO was initiated on the third day of MV. A 17-Fr return cannula was inserted into the right internal jugular vein to a depth of 15 cm. Furthermore, a 21-Fr drainage cannula was inserted into the right femoral vein to a depth of 38 cm. A MAQUET centrifugal pump was employed. Initial ECMO settings included a blood flow rate of 5 L/min, an oxygen concentration of 100%, and a blood-to-gas flow ratio of 1:1. Cannulation and ECMO initiation were uneventful, and SpO_2_ rapidly increased to 100%. Ventilator settings were subsequently reduced to an FiO_2_ of 30%, a PEEP of 5 cmH_2_O, a PC threshold of 10 cmH_2_O, a respiratory rate of 15 breaths/min, and a monitored Vt of 135 mL. During ECMO support, anticoagulation with unfractionated heparin was administered, and APTT was maintained between 50 s and 60 s. Gross hematuria occurred during the night of ECMO initiation, suggesting hemolysis. The condition improved after reducing the blood flow rate of ECMO to 4 L/min.

Sedation was discontinued on ECMO day 10, and the patient regained clear consciousness. MV was discontinued, and oxygen therapy was administered through tracheostomy. Following treatment, both lungs were fully re-expanded. After discontinuation of ECMO sweep gas for 24 h, the patient remained free of chest tightness and dyspnea. Respiratory and hemodynamic conditions were stable, and the ECMO weaning criteria were met. ECMO was successfully discontinued on ECMO day 12. Oxygen was delivered via the tracheostomy tube at a flow rate of 10 L/min, and arterial oxygen tension (PaO_2_) was 121 mmHg. The patient became emotionally agitated following a family visit on the same evening. After straining during defecation, dyspnea worsened again, and SpO_2_ decreased to 80%. Sedation, analgesia, and MV were reinitiated. Additional examinations excluded pulmonary embolism and cardiac insufficiency. The ventilator was set to the P-A/C mode with an FiO_2_ of 80%, a PEEP of 5 cmH_2_O, a PC of 15 cmH_2_O, and a monitored Vt of 380–420 mL. The patient developed purulent airway secretions and fever, and chest imaging suggested secondary bacterial infection. Antimicrobial therapy was escalated to meropenem combined with vancomycin. Air leakage was monitored through the thoracic drainage system after resuming MV, indicating recurrent pneumothorax. Carbon dioxide retention subsequently developed, with the partial pressure of carbon dioxide (PaCO_2_) reaching a maximum value of 78 mmHg. Therefore, V-V ECMO was reinitiated 9 days after removing ECMO. During the second ECMO run, the pneumothorax was not resolved. The patient developed multiple complications, including multidrug-resistant bacterial infection (sputum culture positive for pandrug-resistant *Acinetobacter baumannii*), septic shock, acute renal failure, and drug-induced jaundice. Despite aggressive antibiotic therapy, blood purification therapy, and respiratory and circulatory support, hemodynamic instability persisted. Finally, the patient died of septic shock on day 36 of the second ECMO run, corresponding to hospital day 86.

### 2.2 Case 2

The patient was a 60-year-old man with a height of 175 cm and a body weight of 65 kg who denied a history of homosexual intercourse and intravenous drug use. The patient mentioned a history of hypertension. On December 21, 2024, the patient was admitted to the Department of Infectious Diseases of Beijing Ditan Hospital, Capital Medical University, because of “cough and dyspnea for 1 week, with newly detected positive HIV antibody titers for 1 day.” He had never received ART previously. His CD4^+^ T cell count was 5 cells/μL, and his HIV RNA level was 228,244 copies/mL. Chest CT showed diffuse ground-glass opacities in both lungs. *P. jirovecii* was detected based on sputum PCR, and the patient was diagnosed with AIDS complicated by PCP. After admission, the patient received SMZ-TMP combined with clindamycin as the antibiotic regimen, glucocorticoids, and high-flow nasal oxygen (HFNO) with a gas flow rate of 40 L/min and an oxygen concentration of 55%. ART, consisting of bictegravir sodium, emtricitabine, and tenofovir alafenamide fumarate tablets, was initiated on hospital day 14.

On hospital day 37, the patient developed immune reconstitution inflammatory syndrome (IRIS), accompanied by worsening pulmonary lesions; therefore, he was transferred to the ICU because of respiratory failure. On the day of ICU admission, the P/F ratio was 80 mmHg (1 mmHg = 0.133 kPa), and the respiratory rate was 40 breaths/min. Thus, based on his condition, endotracheal intubation was conducted, and MV was initiated. The ventilator was set to the P-A/C mode with an FiO_2_ of 70%, a PEEP of 6 cmH_2_O (1 cmH_2_O = 0.098 kPa), a PC of 24 cmH_2_O, and a respiratory rate of 22 breaths/min. Static lung compliance was determined to be 20 mL/cmH_2_O. The patient was also complicated by septic shock and received antibiotics, fluid resuscitation, and norepinephrine infusion (0.1 μg/kg/min) for hemodynamic support. On hospital day 51, corresponding to MV day 14, chest CT revealed pneumomediastinum and cervical subcutaneous emphysema; therefore, the patient underwent tracheostomy, and V-V ECMO support was initiated. During ECMO support, anticoagulation with unfractionated heparin was administered, and APTT was maintained between 50 s and 60 s. Oral bleeding and oozing from the cannulation site occurred during ECMO support. The bleeding resolved after reducing the dose of unfractionated heparin and administering a plasma transfusion.

The patient developed a small right-sided pneumothorax on hospital day 85, corresponding to ECMO day 34. Sedation, analgesia, and neuromuscular blockade were intensified. Multiple large thrombi were observed within the ECMO oxygenator, and SpO_2_ decreased. Oxygenator dysfunction was suspected, resulting in difficulty maintaining adequate oxygenation. The patient’s family declined oxygenator replacement. The patient died of respiratory failure on hospital day 89, corresponding to ECMO day 38.

### 2.3 Case 3

Case 3 was a 31-year-old man with a height of 175 cm and a body weight of 52 kg who was previously healthy. The patient noted a history of homosexual intercourse. On August 16, 2025, the patient was admitted to the Department of Infectious Diseases of Beijing Ditan Hospital, Capital Medical University, because of “dyspnea for 10 days, fever for 3 days, and newly detected positive HIV antibody for 1 day.” He had never received ART previously. His CD4^+^ T-cell count was 8 cells/μL, and his HIV RNA level was 4.29^E+5^ copies/mL. Based on the characteristic radiological findings of diffuse ground-glass opacities in both lungs and a positive sputum PCR result for *P. jirovecii*, the patient was diagnosed with AIDS complicated by PCP. After admission, the patient received SMZ-TMP combined with clindamycin to treat infection. Simultaneously, systemic glucocorticoids and oxygen therapy via face mask were also administered. On hospital day 4, the patient was transferred to the ICU due to worsening respiratory failure. On the day of ICU admission, the P/F ratio was 188 mmHg, and the respiratory rate was 25 breaths/min. HFNO was administered with a flow rate of 60 L/min and an oxygen concentration of 60%.

The patient’s dyspnea worsened on hospital day 6, SpO_2_ decreased to 88%, and the respiratory rate increased to 35–40 breaths/min. On physical examination, palpable crepitus was detected on the right side of the neck; thus, endotracheal intubation was conducted, and MV was initiated. The ventilator was set to the volume assist/control (V-A/C) mode with an FiO_2_ of 60%, a PEEP of 6 cmH_2_O, a Vt of 420 mL, and a respiratory rate of 20 breaths/min. Pplat was determined to be 25 cmH_2_O, and static lung compliance was found to be 22 mL/cmH_2_O. Given that the patient required a high level of respiratory support and had concomitant subcutaneous emphysema and a high risk of pneumothorax, V-V ECMO support was initiated on hospital day 9, corresponding to the third day of invasive ventilation. A 15-Fr return cannula was inserted into the right internal jugular vein to a depth of 18 cm. Besides, a 21-Fr drainage cannula was inserted into the right femoral vein to a depth of 48 cm. A MAQUET centrifugal pump was employed. Initial ECMO settings were as follows: a blood flow rate of 4 L/min, an oxygen concentration of 100%, and a blood-to-gas flow ratio of 1:1. Cannulation and ECMO initiation were conducted without adverse events. Subsequently, SpO_2_ rapidly increased to 100%. Ventilator settings were subsequently switched to an FiO_2_ of 40%, a PEEP of 6 cmH_2_O, a PC of 6 cmH_2_O, and a respiratory rate of 15 breaths/min, with a Vt of 230 mL. Postprocedural chest radiography indicated excessive insertion depth of the drainage cannula, with overlap between the drainage and return cannulas, resulting in recirculation. Under ultrasound guidance, the depth of the drainage cannula was adjusted to 45 cm, and the cannula tip was positioned at the junction of the right atrium and inferior vena cava. The depth of the return cannula was adjusted to 16 cm.

Unfractionated heparin was administered as anticoagulation during ECMO support. On ECMO day 5, pulmonary lesions improved, and subcutaneous emphysema resolved. After discontinuing sedation, the patient was fully conscious and had intact cough and swallowing reflexes with adequate airway protection. Following a comprehensive assessment, the endotracheal tube was removed safely. HFNO was provided with a gas flow rate of 40 L/min and an oxygen concentration of 30%. The respiratory rate was maintained at 20–25 breaths/min, and SpO_2_ was kept at 97%. At the same time, ART was initiated, which consisted of bictegravir sodium, emtricitabine, and tenofovir alafenamide fumarate tablets. On ECMO day 10, repeat chest CT showed significant improvement in pulmonary lesions. After discontinuation of the ECMO sweep gas, the patient exhibited no chest tightness or dyspnea. The P/F ratio exceeded 300 mmHg, and respiratory and circulatory conditions stabilized, meeting the ECMO weaning criteria. ECMO was successfully removed on ECMO day 11, and HFNO was continued. On hospital day 26, the patient was transferred out of the ICU, and antibiotic therapy and ART continued in the infectious diseases ward. On hospital day 54, repeat chest CT indicated marked resolution of pulmonary inflammation, and liver and renal function were normal. The patient recovered and was discharged.

### 2.4 Case 4

Case 4 was a 29-year-old man with a height of 180 cm and a body weight of 70.5 kg who had a history of AIDS for more than 3 years and had never received ART. On September 12, 2025, the patient was admitted to the Emergency Department of Beijing Ditan Hospital, Capital Medical University, because of intermittent fever lasting more than 1 month, dyspnea lasting 7 days, and worsening symptoms within 3 days before his hospitalization. His CD4^+^ T-cell count was 20 cells/μL, and his HIV RNA level was 5.99^E+5^ copies/mL. On admission, the P/F ratio of the patient was 49 mmHg, and the respiratory rate was 50 breaths/min; therefore, endotracheal intubation and MV were conducted immediately. Chest radiography on the same day revealed a right-sided pneumothorax with approximately 40% lung compression. Subsequently, right-sided closed thoracic drainage was conducted. After 3 days of emergency treatment, the patient was transferred to the ICU on September 15, 2025. Chest CT showed diffuse ground-glass opacities in both lungs. Sputum PCR detected *P. jirovecii*. The patient was diagnosed with AIDS complicated by PCP and pneumothorax. Treatment included the combination of SMZ-TMP and clindamycin as the anti-infective regimen, glucocorticoids, and MV. The ventilator was set to the P-A/C mode with an FiO_2_ of 60%, a PEEP of 6 cmH_2_O, a PC of 18 cmH_2_O, and a respiratory rate of 16 breaths/min.

The patient required a high level of ventilatory support and had a persistent pneumothorax with continuous air leakage from the drainage system. Due to unsatisfactory resolution of pneumothorax, V-V ECMO was initiated on hospital day 6 (day 3 of invasive ventilation). A 15-Fr return cannula was inserted into the right internal jugular vein to a depth of 15 cm. Furthermore, a 21-Fr drainage cannula was inserted into the right femoral vein to a depth of 45 cm. A MAQUET centrifugal pump was employed for V-V ECMO. Initial ECMO settings included a blood flow rate of 4 L/min, an oxygen concentration of 100%, and a blood-to-gas flow ratio of 1:1. Cannulation and ECMO initiation were conducted without a significant adverse event. Subsequently, SpO_2_ rapidly increased to 100%. Ventilator settings were subsequently modified to an FiO_2_ of 30%, a PEEP of 5 cmH_2_O, a PC of 10 cmH_2_O, a respiratory rate of 15 breaths/min, and a Vt of 200 mL. During ECMO support, anticoagulation was administered with unfractionated heparin.

In the early phase of ECMO support, sedation was maintained with the combination of midazolam and propofol, and analgesia was provided with remifentanil. The Richmond agitation-sedation scale (RASS) score was −4. From ECMO day 6, sedation was gradually reduced, and the RASS score reached −2. After discontinuing the sedative agents, the patient became agitated and could not cooperate with treatment. Awake ECMO was not feasible. The right-sided pneumothorax persisted without healing, and pleurodesis was conducted via autologous blood pleurodesis. On ECMO day 22, pulmonary lesions improved, and ART was initiated, consisting of bictegravir sodium, emtricitabine, and tenofovir alafenamide fumarate tablets. After infection control and hemodynamic stabilization, the patient displayed good spontaneous cough and swallowing function. The endotracheal tube was removed following a comprehensive evaluation. HFNO was applied at a gas flow rate of 50 L/min and an oxygen concentration of 60%. Subsequently, the respiratory rate became 16 breaths/min, and SpO_2_ reached 95%. After extubation, the patient experienced anxiety, agitation, and tachycardia. A high risk of accidental device removal and falls was detected; thus, sedation and analgesia were still required. Tracheostomy was also conducted on the same day, and sedation, analgesia, and MV were continued. Unfractionated heparin was discontinued for 16 h to prevent bleeding after tracheostomy. The patient remained in an anxious state. Psychiatric consultation resulted in the diagnosis of an anxiety disorder; therefore, risperidone and olanzapine were administered. After comprehensive treatment, the right-sided pneumothorax healed, and the drainage tube was removed. The right lung was fully re-expanded, and the pulmonary infection improved. ECMO sweep gas was turned off for 24 h. There was no sign of chest tightness or dyspnea, and the P/F ratio remained more than 300 mmHg with stable respiratory and circulatory function. Additionally, the ECMO weaning criteria were met.

ECMO was successfully removed on ECMO day 27, and MV support was continued. Ventilator settings after ECMO removal were as follows: the P-A/C mode with an FiO_2_ of 30%, a PEEP of 5 cmH_2_O, a PC of 10 cmH_2_O, and a respiratory rate of 16 breaths/min. Five days after ECMO weaning, the patient developed a left-sided pneumothorax with approximately 30% lung collapse. Left-sided closed thoracic drainage was conducted. There was no serious need for respiratory support, and MV was intermittently discontinued. Oxygen therapy was provided via tracheostomy. The left lung re-expanded after adequate drainage. The left-sided pneumothorax resolved 23 days later, and the drainage tube was removed. The patient’s condition improved after 80 days of hospitalization, and the tracheostomy stoma was closed. SpO_2_ was 96% while breathing room air, and the patient was discharged.

## 3 Discussion

PCP is a common opportunistic infection affecting patients with AIDS. A subset of patients with PCP develop severe acute respiratory failure requiring invasive mechanical ventilation. Clinical deterioration occurs in a small proportion of these cases, and mechanical ventilation alone is insufficient to maintain adequate oxygenation, necessitating ECMO support. As a national infectious disease treatment center, Beijing Ditan Hospital, Capital Medical University, has provided V-V ECMO support for four patients with AIDS who were complicated by PCP. The ECMO management process of these patients is summarized below to share clinical experience and improve treatment outcomes.

### 3.1 Indications and timing of ECMO initiation

During the 2009 H1N1 influenza pandemic, multiple observational studies reported that ECMO, as a rescue therapy, can achieve favorable clinical outcomes in patients with severe respiratory failure (7). Subsequently, the international multicenter randomized controlled EOLIA (ECMO to rescue lung injury in severe ARDS) trial was conducted to evaluate the efficacy of early ECMO initiation in adult patients with severe acute respiratory distress syndrome (ARDS) (8). Although the trial did not show a significant decrease in the risk of 60-day mortality, the results require contextual interpretation. The study was conducted more than a decade ago, when ECMO circuit technology, anticoagulation strategies, and multidisciplinary management protocols were substantially less advanced compared to current standards. In addition, a *post hoc* Bayesian analysis performed by Combes et al. suggested that ECMO may significantly reduce mortality (9). More recent studies, including a meta-analysis integrating data from two randomized controlled trials (CESAR and EOLIA, involving a total of 429 patients), have indicated that patients receiving ECMO support exhibit prolonged ICU survival and a lower risk of multiple organ dysfunction, including respiratory, cardiovascular, renal, and neurological failure (10). Therefore, ECMO may prevent mortality and decrease the risk of multiple organ dysfunction in patients suffering from severe respiratory failure.

ECMO should be particularly considered for patients with severe, acute, and potentially reversible respiratory failure who do not respond to conventional modalities of treatment. In irreversible conditions, such as end-stage lung disease, ECMO may serve as a bridge to lung transplantation. The only absolute contraindication to ECMO initiation is the inability to achieve eventual extubation. According to the ELSO guidelines (11), central nervous system hemorrhage, severe central nervous system injury, systemic bleeding, contraindications to anticoagulation, immunosuppression, advanced age (without a defined threshold), mechanical ventilation exceeding 7 days, plateau pressure (Pplat) greater than 30 cmH_2_O, and FiO_2_ greater than 90% represent relative contraindications for ECMO.

It is well recognized that prolonged mechanical ventilation before initiating ECMO is associated with an increased risk of post-ECMO mortality. Patients undergoing mechanical ventilation for 7 days or longer exhibit a 35% reduction in 60-day survival compared with those with early ECMO initiation. In addition, when Pplat exceeds 30 cmH_2_O, and FiO_2_ exceeds 90% for more than 24 h, the likelihood of in-hospital mortality escalates by 2.1-fold (8). Therefore, in patients without contraindications but with clear indications, optimal medical management should be implemented promptly, and ECMO initiation should not be delayed for any reason. The ELSO guidelines recommend that ECMO should be considered for patients with severe ARDS and refractory hypoxemia (PaO_2_/FiO_2_ < 80 mmHg) or severe hypercapnic respiratory failure (pH < 7.25, PaCO_2_ ≥ 60 mmHg) who do not respond to optimal conventional treatments (11).

All four patients admitted to our hospital were diagnosed with AIDS complicated by PCP, and all developed acute respiratory failure. None of the patients had received ART before admission, and all patients presented with markedly reduced CD4^+^ T cell counts, suggesting a profound immunodeficient state. Although immunodeficiency is considered a relative contraindication to ECMO, acute infectious lesions may be curable with adequate antibiotic therapy and organ support. In addition, patients with human immunodeficiency virus (HIV) infection may still achieve immune reconstitution after initiating ART. Therefore, immunodeficiency itself does not represent an absolute contraindication to ECMO. Over recent decades, continuous advances in medical management have significantly improved the survival of patients with acquired immunodeficiency. Early diagnosis and the development of antiretroviral, antibacterial, and antifungal medications have enabled people living with HIV to achieve life expectancy comparable to that of non-HIV-infected individuals. RajSic et al. conducted a systematic review (12) to evaluate the clinical outcomes of patients with newly diagnosed AIDS who were complicated by ARDS and received ECMO support. In total, 288 articles were retrieved, and 22 studies were ultimately included. The results revealed that from 2004 to 2023, 29 HIV-infected patients received ECMO support for refractory ARDS. PCP accounted for 90% of severe ARDS cases. The mean age of participants was 36 ± 13 years. The mean duration of extracorporeal life support was 13 days (7.5–27 days). Among the participating patients, 93% successfully completed ECMO support. During ECMO support, two patients died. Besides, another two died during hospitalization after admission. Although this study was limited by publication bias favoring successful cases, the evidence indicated that ECMO support had no significant adverse effect on HIV-infected patients. In particular, younger patients without underlying chronic lung diseases may achieve a better prognosis with ECMO support. Therefore, ECMO support should not be delayed or withheld in newly diagnosed HIV-infected patients solely due to immunodeficiency.

Among the four cases in our cohort, cases 1 and 2 presented with severe hypoxemia, with persistent PaO_2_/FiO_2_ ratios less than 80 mmHg despite mechanical ventilation. Case 3 required high ventilatory support and developed subcutaneous emphysema, suggesting a high risk of pneumothorax. Case 4 had already developed a pneumothorax. ECMO was initiated to reduce the intensity of mechanical ventilation, thereby preventing ventilator-induced lung injury (VILI) and achieving lung rest and lung protection. ECMO was initiated on day 3, day 14, day 3, and day 6 of mechanical ventilation in the four cases reported in this study.

Case 1 underwent two ECMO runs due to recurrent pneumothorax and finally died of septic shock. His death was deemed to be associated with insufficient early experience in managing severe multidrug-resistant bacterial infections, particularly carbapenem-resistant Gram-negative bacilli. With the development and clinical application of new antimicrobial agents, such as ceftazidime-avibactam and sulbactam-durlobactam, as well as improvements in airway management techniques, the treatment of secondary pulmonary infections caused by multidrug-resistant organisms is expected to improve considerably. Case 2 exhibited persistent non-improving pulmonary lesions and could not be weaned from ECMO due to prolonged support, motivating the family to withdraw treatment. This outcome was attributed to delayed ECMO initiation, older age, and underlying comorbidities. A single-center retrospective study by Marie et al. found that patients older than 65 years were at a higher risk of mortality after ECMO support (13). Therefore, in patients with prolonged mechanical ventilation (>7 days) and advanced age, ECMO is not an absolute contraindication; however, ECMO initiation should be more cautiously evaluated in such cases. Particularly, pulmonary reversibility should be considered to avoid futile treatment.

Cases 3 and 4 achieved favorable outcomes. Specifically, early ECMO support facilitated lung rest, effective antibiotic therapy contributed to the resolution of infection, ART promoted immune reconstitution, intensive organ support partly prevented organ failure and complications. In addition, younger age and absence of comorbidities likely optimized pulmonary recovery and overall prognosis. Case 3 had the shortest ECMO duration, which was attributed to early ECMO initiation. During mechanical ventilation, although plateau pressure was maintained below 30 cmH_2_O, subcutaneous emphysema and pneumomediastinum developed, indicating barotrauma. This may be attributable to pneumatocele formation subsequent to PCP infection. Therefore, in patients with PCP, the risk of barotrauma should be fully assessed. Initiating ECMO before the incidence of pneumothorax may significantly shorten the duration of both ECMO and mechanical ventilation, extend survival, shorten the length of ICU stay, and decrease the risk of secondary infection and other complications.

However, in patients with PCP who have already developed pneumothorax, ECMO combined with closed thoracic drainage appears to be the only feasible therapeutic option, as pneumothorax caused by PCP is extremely difficult to resolve. In the future, the evaluation of ECMO initiation timing will likely shift from a sole focus on gas exchange impairment to a more comprehensive assessment of VILI, including its severity and systemic consequences. Hypoxemia associated with PCP is often refractory to conventional treatments and is susceptible to complications, such as spontaneous pneumothorax and barotrauma, during mechanical ventilation. Therefore, early initiation of ECMO support before the incidence of severe barotrauma is strongly recommended.

### 3.2 Selection of the ECMO support modality

The ECMO support modality should be flexibly selected according to the organ system requiring support. For patients with isolated respiratory failure and preserved cardiac function, V-V ECMO is the preferred modality. V-V ECMO provides only gas exchange support, while systemic perfusion relies on the native cardiac pump function. All four patients in our center suffered from respiratory failure with preserved cardiac function; therefore, V-V ECMO was selected in all cases. In the meta-analysis conducted by RajSic et al. among 29 patients, two received veno-arterial (V-A) ECMO due to cardiopulmonary resuscitation or heart failure (12).

### 3.3 Management of mechanical ventilation during ECMO support

After initiating V-V ECMO, a more stringent lung-protective ventilation strategy is emphasized, which can reduce VILI, attenuate inflammatory responses, and improve prognosis. As early as 2009, Terragni et al. found that among patients with ARDS who were ventilated with a tidal volume of 6 mL/kg ideal body weight (IBW), 30% still exhibited overdistension. The incidence of overdistension decreased when tidal volume was reduced to 4–4.5 mL/kg IBW through extracorporeal carbon dioxide removal (14). In 2016, Terragni et al. conducted a meta-analysis including nine studies and 545 patients. The study revealed that in patients with ARDS receiving ECMO for refractory hypoxemia, driving pressure during ECMO support was the only ventilatory parameter independently associated with in-hospital mortality (15). Subsequently, multiple clinical studies have shown the feasibility of an ultraprotective ventilation strategy using tidal volumes of 3–4 mL/kg IBW. This strategy is defined by a tidal volume of ≤4 mL/kg IBW, a respiratory rate of <20 breaths/min, a plateau pressure (Pplat) of <25 cmH_2_O, and a driving pressure of ≤15 cmH_2_O (11). Optimal management of ventilation in ECMO-supported patients may require balancing low driving pressure, adequate lung inflation, and low respiratory rate (16).

Compared with patients with ARDS and normal immune function, patients with PCP exhibit distinct pathological characteristics of the lung. PCP-related lung injury comprises diffuse alveolar damage, interstitial fibrosis, and characteristic cystic destruction of lung parenchyma. The latter leads to fragile alveolar walls that are highly susceptible to air leak syndromes, including spontaneous pneumothorax and pneumomediastinum. In addition, HIV-infected patients may develop IRIS after initiating ART. IRIS is characterized by an excessive inflammatory response during immune recovery, which may lead to abrupt deterioration of respiratory function. This was the main cause of exacerbating respiratory failure in case 2. ECMO plays a dual role in this context. On one hand, it ensures gas exchange and provides a time window for ART initiation and immune recovery. On the other hand, it enables a minimal ventilation “lung rest” strategy to limit secondary inflammatory damage to the lung. Under ECMO support, lung rest ventilation typically adopts moderate to low PEEP levels (8–14 cmH_2_O) to maintain functional residual capacity and reduce shear stress caused by repetitive alveolar opening and collapse. PEEP settings must be carefully individualized among patients with AIDS and PCP who are complicated by cystic lung lesions. Excessively high PEEP may exacerbate alveolar rupture and increase the risk of air leakage. Therefore, under the premise of maintaining adequate oxygenation, relatively lower PEEP targets should be considered, with priority given to the prevention of further lung injury. However, the evidence base for ventilatory management of ECMO in patients with AIDS remains limited, and data mainly come from small case series, registry studies, and expert opinion (17–19). Specifically, there are no prospective randomized controlled trials in this regard. ECMO ventilator settings should be individualized based on dynamic respiratory mechanics monitoring instead of adopting a uniform fixed strategy for all patients.

Before ECMO weaning, ventilatory management gradually transitions from a lung rest strategy to conventional ventilation. With improvements in pulmonary function, tidal volume can be gradually increased to approximately 6 mL/kg IBW, while ECMO blood flow is often reduced to assess the stability of spontaneous breathing and gas exchange. It is important to emphasize that pulmonary recovery does not guarantee complete pulmonary healing. After ECMO decannulation, lung-protective ventilation with low driving pressure should still be maintained.

### 3.4 Sedation and analgesia in ECMO support

Analgesia, sedation, and neuromuscular blockade aim to alleviate patient stress, prevent agitation, and improve patient–ventilator synchrony. Early and adequate sedation and analgesia also help reduce excessive inspiratory effort and decrease excessive transpulmonary pressures. Excessive transpulmonary pressures may intensify lung stress and strain, thereby preventing or mitigating the incidence and progression of lung injury. Neuromuscular blockade should be added when sedation and analgesia alone are insufficient to eliminate excessive inspiratory effort. With clinical improvement, sedation can be gradually reduced, allowing progressive awakening, and even discontinuation of MV with the removal of the endotracheal tube. Awake ECMO can be implemented in such cases (20). Awake ECMO is particularly beneficial in patients with pneumothorax, as it facilitates healing. Case 3 represented a typical successful application of awake ECMO. Pulmonary lesions rapidly improved after ECMO support combined with lung rest and antibiotic therapy. The patient cooperated well after sedation withdrawal, and barotrauma did not further progress. ICU length of stay was significantly reduced, and the prognosis was favorable. In contrast, due to psychological factors, case 4 experienced anxiety and agitation after sedation withdrawal and could not cooperate with treatment. This resulted in recurrent pneumothorax, and awake ECMO could not be successfully implemented at an early stage. After treating anxiety and depression, combined with intensified sedation and analgesia, the pneumothorax eventually resolved, and the patient was discharged after clinical improvement. However, ECMO duration, hospital stay, ICU stay, and overall treatment costs all increased.

## 4 Conclusion

As a short-term and effective mechanical support modality that replaces cardiopulmonary function, ECMO has shown definite efficacy in the treatment of ARDS. However, there are currently no reports on the application of ECMO in Chinese patients with AIDS, and only a few international reports exist in this regard (12). This is mainly because immunosuppression is considered a relative contraindication to ECMO (12); thus, it is not routinely used for patients with HIV infection. In our center, V-V ECMO was used to treat patients with AIDS complicated by respiratory failure. Among the patients, two were successfully discharged. Therefore, ECMO may be considered a salvage therapy in such patients. Immunodeficiency alone should not be regarded as a reason for delaying or withholding ECMO support. In particular, for patients at high risk of pneumothorax or those who have already developed pneumothorax, ECMO should be initiated early after the exclusion of contraindications. Clinicians should fully understand the support capabilities of different ECMO modes, comprehensively evaluate cardiopulmonary function, select appropriate support strategies, implement refined management strategies for sedation and analgesia, and combine antibiotics and other types of supportive care. Prior to weaning, careful assessment remains pivotal to improve survival outcomes.

## Data Availability

The original contributions presented in the study are included in the article/supplementary material, further inquiries can be directed to the corresponding author.
